# Heparin-Binding Protein in Critically Ill Children With Severe Community-Acquired Pneumonia

**DOI:** 10.3389/fped.2021.759535

**Published:** 2021-10-28

**Authors:** Caizhi Huang, Cong Zhang, Jie Zhang, Lin Zhang, Yi Mo, Liya Mo

**Affiliations:** Department of Laboratory Medicine, Hunan Children's Hospital, Changsha, China

**Keywords:** heparin-binding protein, respiratory failure, sepsis, community-acquired pneumonia, children

## Abstract

**Objective:** The aim of this study was to investigate possible associations between Heparin-binding protein (HBP) and the development of respiratory failure (RF) and sepsis in critically ill children with severe community-acquired pneumonia (CAP).

**Methods:** This study enrolled 157 children with severe CAP admitted to Intensive Care Unit (ICU). At ICU admission, the levels of HBP and other biomarkers, including C-reactive protein, interleukin-6 (IL-6), procalcitonin, white blood cells, neutrophil percentage, and D-dimer, were determined.

**Results:** Of the enrolled patients, 106 developed RF (35 with RF at enrollment and 71 with RF after enrollment), while 51 did not developed RF. The number of patients progressing to sepsis in those with or without RF were 34 (21 with severe sepsis) and 14, respectively. The plasma level of HBP at admission was more than eightfold higher than the upper normal value. HBP, IL-6, and D-dimer could significantly predict the development of RF, and a high level of HBP (odds ratio = 1.008, 95% confidence interval: 1.003–1.013) was independently associated with the development of RF in this population. Compared with other biomarkers, HBP was the best indicator of progression to severe sepsis, with an area under the receiver operating characteristic curve of 0.85, the best specificity at 96.30%, and a positive predictive value of 92.86% at the optimal cut-off value of 340.29 ng/mL. The HBP level was also positively correlated with other conventional biomarkers.

**Conclusion:** HBP might represent a better predictor of disease progression in children with severe CAP than currently used biomarkers.

## Introduction

Community-acquired pneumonia (CAP) is one of the most common pediatric infectious diseases and remains the most common cause of death in children below 5 years of age (1). The World Health Organization estimates that more than 800,000 children under the age of 5 years died of CAP in 2017, accounting for 15% of all deaths in this age group, with most of these deaths occurring in developing countries. Severe CAP is a life-threatening condition that requires hospitalization. Early prediction and accurate assessment of disease progression and prognosis are essential for clinical decision-making in children with severe CAP. However, in everyday clinical practice, severe CAP is frequently associated with both diagnostic and therapeutic uncertainties due to the use of antibiotics prior to admission and the low specificity of signs and symptoms.

It is well known that CAP is an acute lung infection caused by various pathogens. CAP is immunopathologically characterized by an inflammatory response in the lower respiratory tract, which is regulated by cytokines and other inflammatory mediators. These biomarkers reflect a host's response to infection and may provide an objective measure of disease progression that, when regularly monitored, can improve the prognosis of children with CAP. Some well-known biomarkers, including white blood cell (WBC) count, absolute neutrophil count, C-reactive protein (CRP), interleukin-6 (IL-6) and procalcitonin (PCT), are involved in the identification of clinical conditions of CAP (2–4). Nevertheless, data about the association of biomarkers with disease severity in children with CAP remain limited. Some studies have reported that conventionally measured biomarkers are generally not useful for predicting illness severity in CAP children (5). Thus, it is necessary to identify and explore novel biomarkers to better understand their associations with specific clinical situations in children with CAP, as well as to improve treatment options.

Heparin-binding protein (HBP), also known as azurocidin or cationic antimicrobial protein of 37 kDa, is a multifunctional immunomodulatory protein contained within the secretory and azurophilic granules of neutrophilic granulocytes. HBP is rapidly released upon stimulation of leukocytic membrane-bound β_2_-integrins. HBP has several properties that contribute to the inflammatory process, including a broad spectrum of antimicrobial activities, potent chemoattractant and activator effects on monocytes and macrophages, and the ability to increase vascular permeability with consequent edema formation (6). Elevated plasma levels of HBP have been found to be associated with severe infection, organ failure, and mortality in critically ill adult patients (7–11). However, there are scant data currently available related to the relationship between HBP and CAP in children. Therefore, in the present study, we aimed to analyze the admission levels of plasma HBP and other conventional biomarkers, including CRP, IL-6, PCT, WBC, neutrophil percentage (N%), and D-dimer, and to explore possible associations between HBP levels and the development of respiratory failure (RF) and sepsis in critically ill children with severe CAP.

## Materials and Methods

### Study Population

The protocol conformed to the provisions of the Declaration of Helsinki and the study was approved by the Ethics Committee of Hunan Children's Hospital. Informed consent that medical records might be collected and used for clinical research was obtained from the legal guardians of all individuals at admission in this study. The definition of CAP and severity assessments were in accordance with the British Thoracic Society 2011 guidelines for the management of CAP in children (2). RF was defined as a PaO_2_ less than 50 mm Hg at any time or the need for mechanical ventilation. Sepsis, severe sepsis, and septic shock were defined according to International Pediatric Sepsis Consensus Conference (12).

This retrospective, observational study involved a consecutive sample of children admitted to the intensive care unit (ICU) of Hunan Children's Hospital from October 2019 to September 2020 with severe CAP. Exclusion criteria included an ICU stay of <24 h; the absence of informed consent or measurement data; ventilator associated pneumonia or hospital acquired infections; or a hematological condition, immunocompromised state, or other chronic medical conditions, such as congenital heart disease, chronic lung disease, or a primary abnormality of coagulation. A total of 287 children with severe CAP were initially enrolled. After application of these exclusion criteria, 130 children were excluded, and 157 children were included in further evaluations.

**Figure 1:**
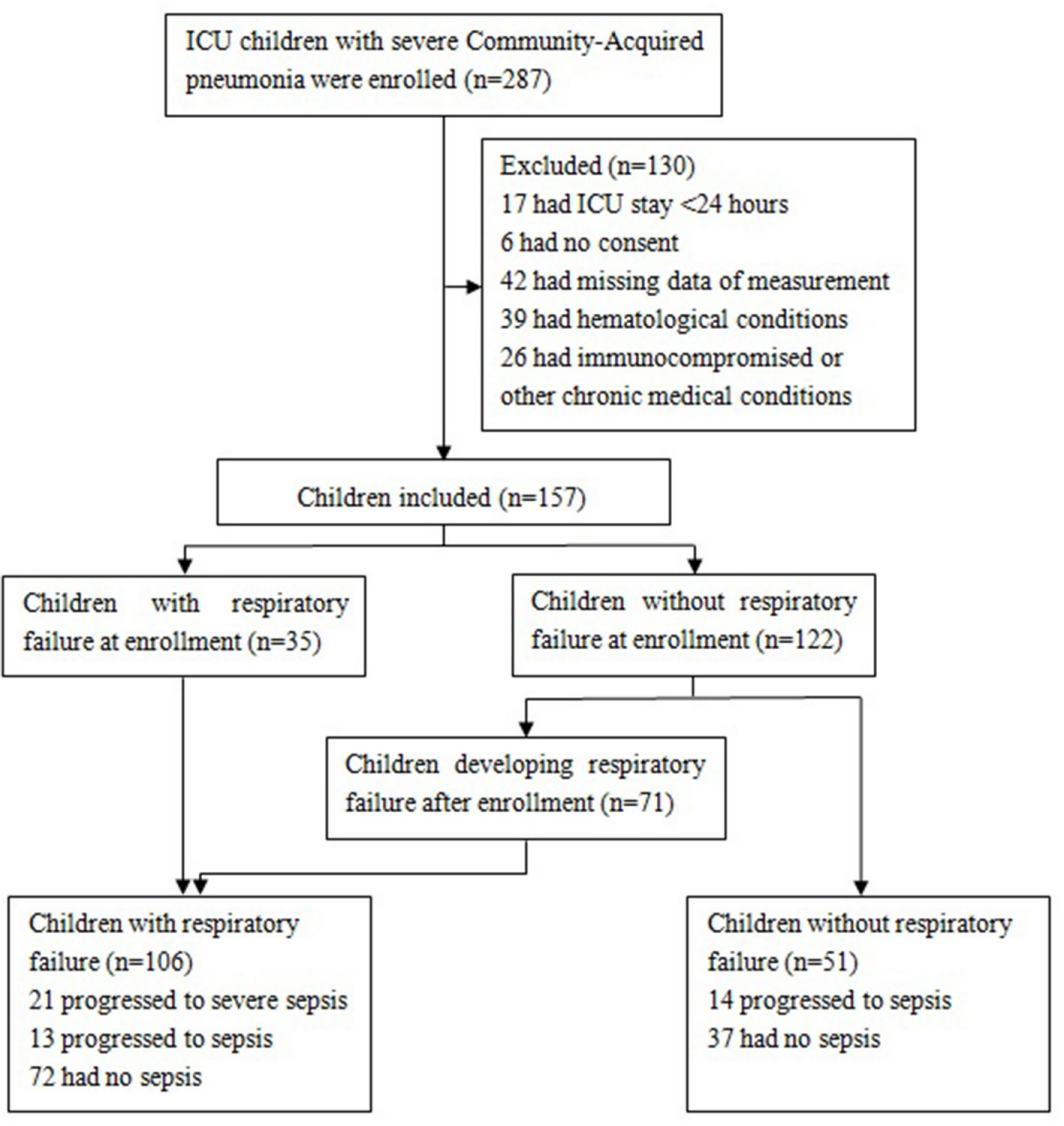
Flowchart of participants. Patients with severe sepsis included those with septic shock (*n* = 10) and those without septic shock (*n* = 11).

The included patients were classified into the following groups based on the presence or absence of RF: (a) CAP with RF (*n* = 106), including 35 with RF at enrollment and 71 who developed RF after enrollment, and (b) CAP without RF (*n* = 51). In the former group, 21 children progressed to severe sepsis (with or without septic shock), and 13 children progressed to sepsis. In the latter group, 14 children progressed to sepsis. The flowchart of children in the study is shown in Figure 1.

### Data Collection

In all participants, blood samples for detection of HBP were collected immediately after ICU admission. Samples were collected into sodium citrate tubes and were centrifuged within the next 30 min. The examination of plasma HBP was performed within two h. HBP was assayed by a dry quantitative immunofluorescence assay (Jet–iStar 3000; JoinStar, Hangzhou, China) according to the manufacturer's recommendations. The lower detection limit was 5.9 ng/mL for HBP, and the normal reference range for HBP was defined as below 11.40 ng/mL. The plasma level of HBP was determined while blinded to the study protocol. Other candidate biomarkers, including CRP, IL-6, PCT, WBC, N%, and D-dimer were also measured and recorded for further analyses. The Laboratory Medicine Department of Hunan Children's Hospital provided all blood tests.

### Statistical Analysis

Data were analyzed using SPSS version 19.0 software. The normality test found that all biomarkers levels (i.e., HBP, CRP, IL-6, PCT, WBC, N%, and D-dimer) were not normally distributed (*P* < 0.01). Thus, nonparametric analyses were used for all data. The measurement data are presented as medians (interquartile ranges) and were compared using either the Kruskal–Wallis *H* test or the Mann–Whitney *U* test, as appropriate. Comparisons between multiple groups were performed using the Kruskal–Wallis *H* test followed by rank transformation of the least-significant difference test. Numerical data are presented as numbers and percentages (%) and were compared using the chi-squared test. Areas under the receiver operating characteristic (ROC) curves were calculated to evaluate the predictive power of each biomarker. The Spearman rank correlation test was used to analyze relationships between two continuous variables. Univariate and multivariate logistic regression analyses were performed to identify independent predictors of RF. All probabilities were two-tailed, and *P-*values below 0.05 were regarded as statistically significant.

## Results

### Patient Characteristics

Details on the characteristics of the study population are presented in Table 1. Compared with patients without RF, patients with RF had a younger age; higher rate of mechanical ventilation; longer length of ICU stay; and higher levels of blood HBP, IL-6, and D-dimer at admission. In all CAP children (*n* = 157), HBP had a median level of 98.09 ng/mL, which was more than eightfold higher than the upper normal value of 11.40 ng/mL. The median levels of other biomarkers, including CRP, IL-6, PCT, WBC, N%, and D-dimer, were 12.30 mg/L, 22.60 pg/mL, 0.29 ng/mL, 10.20 × 10^9^/L, 61.60%, and 0.79 μg/mL, respectively. In the 157 children with severe CAP, 65 (41.40%) had positive results on a bacterial culture, and 125 (79.62%) were administered antibiotics before admission.

**Table 1:** Characteristics of the study population.

**Characteristic**	**Without respiratory failure (*n =* 51)**	**Respiratory failure (*n =* 106)**	***P* value**
Age, months	22.00 (10.00–53.00)	9.00 (3.00–29.00)	0.001
Male, *n* (%)	35 (68.63)	62 (58.49)	0.221
Mechanical ventilation, *n* (%)	0 (0)	29 (27.36)	<0.001
Developing sepsis, *n* (%)	14 (27.45)	34 (32.08)	0.556
Length of ICU stay, days	3 (3–5)	8 (5–14)	<0.001
ICU mortality, n (%)	0 (0)	8 (7.55)	0.104
Antibiotic use before enrollment, n (%)	41 (80.39)	84 (79.25)	0.867
Positive bacterial culture (blood), *n* (%)	2 (3.92)	6 (5.66)	0.939
Positive bacterial culture (sputum), *n* (%)	16 (31.37)	49 (46.23)	0.077
Laboratory findings on admission			
HBP, ng/mL	62.07 (28.32–109.80)	113.13 (56.43–214.30)	<0.001
CRP, mg/L	8.91 (1.48–31.70)	12.70 (2.06–44.95)	0.229
IL-6, pg/mL	17.27 (6.37–32.50)	33.60 (12.66–104.69)	<0.001
PCT, ng/mL	0.22 (0.10–0.56)	0.33 (0.09–1.43)	0.306
WBC, 10^9^/L	9.79 (6.49–13.94)	10.59 (7.15–13.83)	0.399
N%	55.60 (35.00–73.00)	64.10 (43.33–77.23)	0.164
D-dimer, μg/mL	0.62 (0.42–1.03)	0.91 (0.52–4.02)	0.007

*Measurement data were presented as medians (interquartile ranges) and analyzed by the Mann–Whitney U test. Numerical data were presented as numbers and percentages (%) and analyzed by the chi-squared test. ICU, intensive care unit; HBP, heparin-binding protein; CRP, C-reactive protein; IL-6, interleukin-6; PCT, procalcitonin; WBC, white blood cell count; N%, neutrophil percentage*.

### Association of HBP and Other Biomarkers With Development of RF

**Figure 2:**
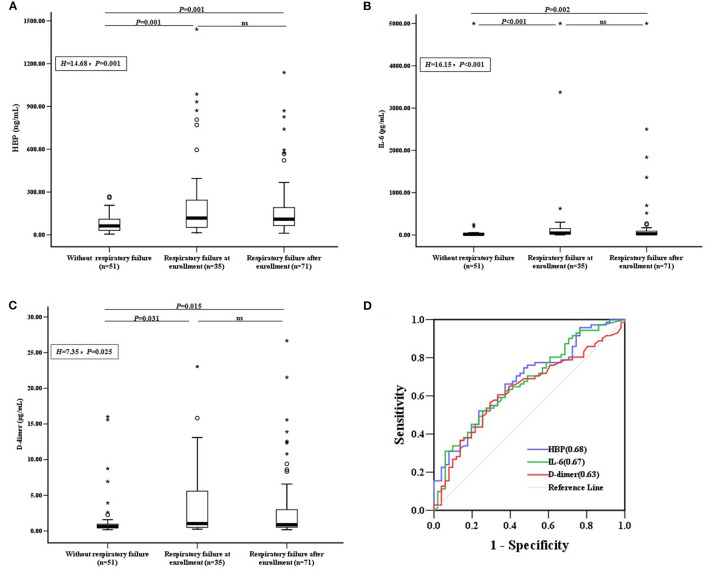
Biomarkers and the development of RF. **(A)** HBP, **(B)** IL-6, and **(C)** D-dimer levels were compared between CAP children with RF at enrollment, with RF after enrollment, and without RF. Differences between these groups were analyzed using the Kruskal–Wallis *H* test, followed by rank transformation of the least-significant difference test. **(D)** ROC curves for biomarkers predicting the development of RF in children without RF at enrollment (*n* = 122), and AUCs are indicated on the graph. AUC, area under the receiver operating characteristic curve; HBP, heparin-binding protein; IL-6, interleukin-6; ns, not significant; ROC, receiver operating characteristic; RF, respiratory failure; °outlier; *extreme.

The levels of CRP, PCT, WBC, and N% were not significantly different between the groups of CAP children with RF at enrollment, with RF after enrollment, and without RF (*P* > 0.05). However, the levels of HBP, IL-6, and D-dimer were significantly lower in children without RF than in the other two groups. The HBP, IL-6, and D-dimer levels did not significantly differ between children who developed RF at enrollment vs. after enrollment (*P* > 0.05) (Figures 2A–C).

To assess whether candidate biomarkers could identify patients who developed RF, data from the 122 CAP children without RF at enrollment were analyzed. Of these, 71 (58.20%) developed RF within the study period. The predictive power for identifying children who developed RF was significant for HBP, IL-6, and D-dimer, with areas under the receiver operating characteristic curves (AUC) of 0.68, 0.67, and 0.63, respectively (Figure 2D). The other markers, including CRP, PCT, WBC, and N% could not significantly predict the development of RF (*P* > 0.05).

Our univariate analysis showed that patients with severe CAP who developed RF after enrollment had a younger age and higher levels of HBP, IL-6, and D-dimer than severe CAP patients who did not develop RF. After adjusting for the confounding variable of age, the multivariate logistic regression analysis demonstrated that a high level of HBP (odds ratio [OR] = 1.008, 95% confidence interval [CI]: 1.003–1.013) was independently correlated with the development of RF in children with severe CAP (Table 2).

**Table 2:** Univariate and multivariate analyses of respiratory failure in children with severe CAP.

**Factor**	**Without respiratory failure (*n =* 51)**	**Respiratory failure after enrollment (*n =* 71)**	**Univariate analysis**		**Multivariate analysis**	
			**z/χ^2^ value**	***P* value**	**OR (95% CI)**	***P* value**
Age, months	22.00 (10.00–53.00)	10.00 (3.00–33.00)	2.78	0.005	0.985 (0.973–0.998)	0.018
Male, n (%)	35 (68.63)	42 (59.15)	1.14	0.285		
HBP, ng/mL	62.07 (28.32–109.80)	110.22 (62.24–192.54)	3.32	0.001	1.008 (1.003–1.013)	0.003
CRP, mg/L	8.91 (1.48–31.70)	12.50 (2.11–44.70)	1.06	0.291		
IL-6, pg/mL	17.27 (6.37–32.50)	32.30 (12.30–95.60)	3.16	0.002	0.999 (0.999–1.000)	0.217
PCT, ng/mL	0.22 (0.10–0.56)	0.30 (0.08–1.29)	0.57	0.570		
WBC, 10^9^/L	9.79 (6.49–13.94)	10.93 (7.26–15.96)	1.33	0.185		
N%	55.60 (35.00–73.00)	63.60 (43.40–76.20)	1.23	0.219		
D-dimer, μg/mL	0.62 (0.42–1.03)	0.87 (0.53–3.26)	2.44	0.015	1.100 (0.952–1.271)	0.198

*Measurement data were presented as medians (interquartile ranges) and analyzed by the Mann–Whitney U test. Numerical data were presented as numbers and percentages (%) and analyzed by the chi-squared test. Statistically significant factors identified in the univariate analysis were further analyzed using a binary multivariate logistic regression analysis. CAP, community-acquired pneumonia; HBP, heparin-binding protein; CRP, C-reactive protein; IL-6, interleukin-6; PCT, procalcitonin; WBC, white blood cell count; N%, neutrophil percentage; OR, odds ratio; 95% CI, 95% confidence interval*.

### Predictive Value of HBP and Other Biomarkers for Sepsis and Severe Sepsis

**Figure 3:**
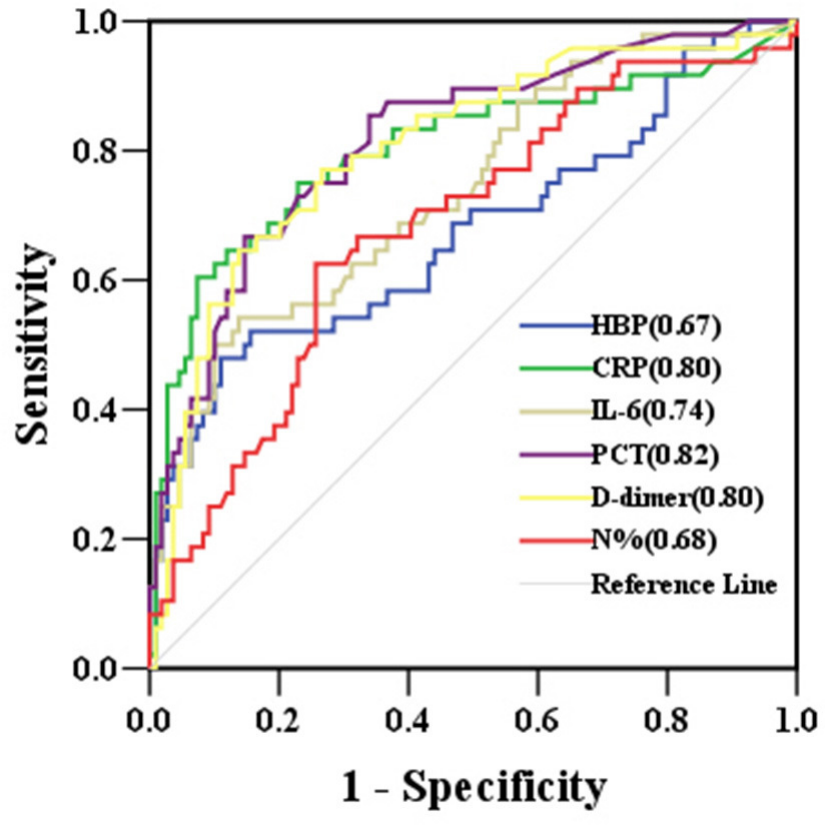
ROC curves for biomarkers predicting the development of sepsis in children with severe community-acquired pneumonia. AUCs are indicated on the graph. AUC, area under the receiver operating characteristic curve; HBP, heparin-binding protein; CRP, C-reactive protein; IL-6, interleukin-6; PCT, procalcitonin; N%, neutrophil percentage; ROC, receiver operating characteristic.

Of the 157 children with severe CAP, 48 developed sepsis, including 21 with severe sepsis (10 with septic shock and 11 without septic shock). The number of sepsis cases was not significantly different between children with or without RF (*P* > 0.05). However, children with sepsis had higher levels of HBP, CRP, IL-6, PCT, N%, and D-dimer than children without sepsis. The predictive ability to identify children who progressed to sepsis was significantly moderate for PCT (AUC 0.82), D-dimer (AUC 0.80), CRP (AUC 0.80), and IL-6 (AUC 0.74) and was significantly mild for N% (AUC 0.68) and HBP (AUC 0.67). WBC was not significant for predicting children who progressed to sepsis (*P* > 0.05) (Table 3 and Figure 3).

**Table 3:** Biomarkers in children with or without sepsis.

**Biomarker**	**Without sepsis (*n =* 109)**	**Sepsis (*n =* 48)**	***Z***	***P***
HBP (ng/mL)	89.95 (41.71–126.84)	151.81 (61.61–490.41)	3.47	0.001
CRP (mg/L)	5.00 (1.48–18.84)	63.10 (18.18–128.83)	6.03	<0.001
IL-6 (pg/mL)	19.50 (7.46–42.48)	64.89 (19.05–252.03)	4.81	<0.001
PCT (ng/mL)	0.16 (0.08–0.43)	1.02 (0.38–10.73)	6.32	<0.001
WBC (10^9^/L)	9.66 (6.77–13.56)	11.70 (7.62–16.41)	1.53	0.125
N%	55.80 (34.30–72.65)	72.50 (54.18–82.95)	3.63	<0.001
D-dimer (μg/mL)	0.61 (0.38–1.05)	3.29 (0.93–10.64)	6.06	<0.001

*Measurement data were presented as medians (interquartile ranges) and analyzed by the Mann–Whitney U test. HBP, heparin-binding protein; CRP, C-reactive protein; IL-6, interleukin-6; PCT, procalcitonin; WBC, white blood cell count; N%, neutrophil percentage*.

**Figure 4:**
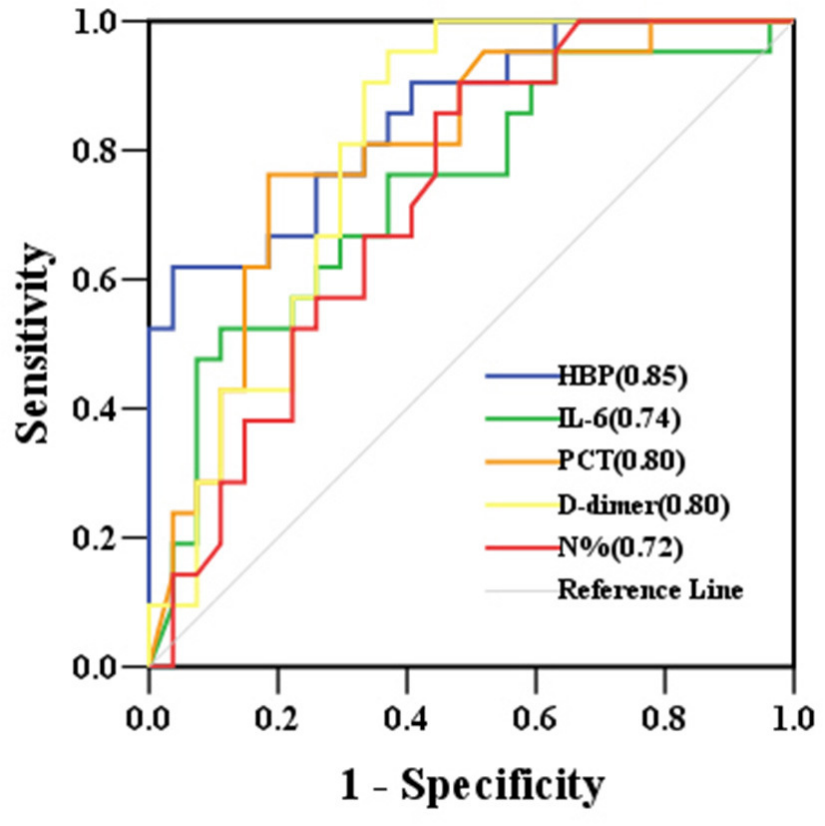
ROC curves for biomarkers predicting the development of severe sepsis in children with sepsis. AUCs are indicated on the graph. AUC, area under the receiver operating characteristic curve; ROC, receiver operating characteristic curve; HBP, heparin-binding protein; IL-6, interleukin-6; PCT, procalcitonin; N%, neutrophil percentage.

The levels of HBP, IL-6, PCT, N%, and D-dimer were significantly higher in the severe sepsis group than in the sepsis group (Table 4). The HBP level (AUC 0.85) had the highest predictive power for identifying children who developed severe sepsis, followed by D-dimer (AUC 0.80), PCT (AUC 0.80), IL-6 (AUC 0.74), and N% (AUC 0.72). At its optimal cut-off value of 340.29 ng/mL, HBP had the best specificity at 96.30% and a positive predictive value of 92.86%. At the optimal cut-off value of 1.94 μg/mL, D-dimer had the best sensitivity at 95.20% and a negative predictive value of 94.44% (Table 5 and Figure 4). The combination of HBP and D-dimer had higher predictive power (AUC 0.86) than any single biomarker for identifying children who developed severe sepsis, with specificity of 88.90% and sensitivity of 71.40%. Neither the CRP level nor the WBC count was significantly different between the sepsis and severe sepsis groups (*P* > 0.05).

**Table 4:** Biomarkers in children with sepsis or severe sepsis.

**Biomarker**	**Sepsis (*n =* 27)**	**Severe sepsis (*n =* 21)**	***Z***	***P***
HBP (ng/mL)	93.41 (34.57–194.55)	574.40 (154.61–848.30)	4.17	<0.001
CRP (mg/L)	44.70 (7.69–97.08)	63.30 (36.04–148.64)	1.63	0.103
IL-6 (pg/mL)	35.20 (17.27–123.10)	202.93 (42.50–662.04)	2.82	0.005
PCT (ng/mL)	0.46 (0.30–1.83)	4.36 (1.48–29.38)	3.50	<0.001
WBC (10^9^/L)	12.22 (7.72–17.39)	9.99 (6.69–14.34)	1.26	0.209
N%	62.00 (45.80–76.70)	78.50 (69.90–86.10)	2.64	0.008
D-dimer (μg/mL)	0.97 (0.64–6.18)	6.94 (3.29–12.73)	3.54	<0.001

*Measurement data were presented as medians (interquartile ranges) and analyzed by the Mann–Whitney U test. HBP, heparin-binding protein; CRP, C-reactive protein; IL-6, interleukin-6; PCT, procalcitonin; WBC, white blood cell count; N%, neutrophil percentage*.

**Table 5:** Performance characteristics of biomarkers for predicting severe sepsis.

**Biomarker**	**Cut-off value**	**AUC**	***P***	**95% CI**	**Sensitivity (%)**	**Specificity (%)**	**PPV (%)**	**NPV (%)**
HBP	340.29 ng/mL	0.85	<0.001	0.748–0.960	61.90	96.30	92.86	76.47
IL-6	187.37 pg/mL	0.74	0.005	0.595–0.883	52.40	88.90	78.57	70.59
PCT	1.95 ng/mL	0.80	<0.001	0.669–0.925	76.20	81.50	76.19	81.48
N%	64.40	0.72	0.008	0.581–0.867	90.50	51.90	59.38	87.50
D-dimer	1.94 μg/mL	0.80	<0.001	0.675–0.927	95.20	63.00	66.67	94.44

*Cut-off values were determined at the maximum values of Youden's index. AUC, area under the receiver operating characteristic curve; 95% CI, 95% confidence interval; PPV, positive predictive value; NPV, negative predictive value; HBP, heparin-binding protein; IL-6, interleukin-6; PCT, procalcitonin; N%, neutrophil percentage*.

### Relationship Between Level of HBP and Other Candidate Biomarkers

The Spearman rank correlation test showed that plasma levels of HBP were positively correlated with levels of N%, IL-6, CRP, D-dimer, PCT, and WBC (*r* = 0.682, 0.419, 0.411, 0.336, 0.327, 0.283, respectively, *P* < 0.001).

## Discussion

In this study, plasma levels of HBP were investigated in children with severe CAP at ICU admission and were compared with other conventional biomarkers. The present study indicated that the median level of HBP at ICU admission was more than eightfold higher than the upper normal value in children with severe CAP. Additionally, higher levels of HBP were correlated with severe sepsis and were independently associated with the development of RF. The HBP level also had a relatively close correlation with the N%.

RF is one of the most serious complications of severe CAP. Although the pathophysiology of RF in patients with critical illness has not been fully determined, activated leukocytes seem to play an important role by changing the vascular permeability, which contributes to respiratory dysfunction. During a lung infection, the inflammatory response can disrupt capillary endothelial and alveolar epithelial barriers and increase the permeability of the microvasculature and alveoli, resulting in pulmonary edema and the development of RF. HBP, which is prefabricated and released from the granules of activated neutrophils, has the ability to induce cytoskeletal rearrangement and to enhance the permeability of endothelial cells by interacting with luminal glycosaminoglycans and by activating the protein kinase C and Rho-kinase pathways (13).

Previous studies have shown that HBP is associated with a low oxygenation ratio and the development of acute lung injury and RF (13–15). In the present study, the levels of HBP, IL-6, and D-dimer in children with RF at or after enrollment were significantly higher than in children without RF. After adjusting for the confounding variable of age, the multivariate logistic regression analysis revealed that the increased level of HBP was independently associated with the development of RF in children with severe CAP. These results are mainly concordant with previous studies and indicate that an increased plasma HBP level is not a consequence of RF, but rather a preceding event.

HBP, with its antimicrobial and proinflammatory activities; its ability to induce vascular leakage and to chemoattract and activate neutrophils, monocytes, and T-lymphocytes; and its mediation of the inflammatory response, is considered to be a predictor of sepsis and is associated with the pathophysiology of organ dysfunction in sepsis (16, 17). In this study, several inflammatory biomarkers, including HBP, CRP, IL-6, N%, PCT, and D-dimer, could significantly predict sepsis. However, the ability of HBP to predict progression to sepsis in children with severe CAP was inferior to PCT, D-dimer, CRP, IL-6, and N%. These results are inconsistent with the findings of another study in adult patients (18), in which it was demonstrated that the AUC of HBP for predicting sepsis was higher than those of PCT or CRP. This discrepancy may result from differences in the study populations, underlying conditions, pathogenic species, antibiotic therapies before enrollment, time of sample collection, and other unknown reasons, indicating that use of HBP in various routine clinical scenarios warrants further investigation.

However, when used to predict progression to severe sepsis (with or without septic shock) in children who already had sepsis, the HBP level had the highest AUC value. At its optimal cut-off value of 340.29 ng/mL, HBP had the best specificity and positive predictive value, while D-dimer had the best sensitivity and negative predictive value at its optimal cut-off value of 1.94 μg/mL. These results suggest that currently used sepsis biomarkers (IL-6, PCT, CRP, N%, and D-dimer) are useful in the early diagnosis of sepsis; however, they are not as useful for evaluating severity in critically ill children. In these cases, HBP seemed to be superior. Therefore, the combination of HBP with other biomarkers, such as D-dimer, may be a more optimal option for routine clinical use. These findings support a previous study conducted by Linder et al. (8).

In the present study, plasma levels of HBP were positively correlated with the levels of N%, IL-6, CRP, D-dimer, PCT, and WBC, suggesting that HBP and other candidate mediators increase in response to infection and inflammation. Since HBP originates from neutrophils, it has a relatively close correlation with the N% (*r* = 0.682). It was also found that children with severe CAP already exhibited a high median HBP level (98.09 ng/ml) at ICU admission. Additionally, the optimal cut-off value of HBP (340.29 ng/mL) for identifying severe sepsis was much higher than the values suggested in previous studies in adult patients (≥30 ng/mL for severe sepsis and ≥ 103.5 ng/mL for septic shock) (16, 18). This discrepancy may result from differences in disease spectrum and course, patient cohorts, blood collection and detection methods, and responses to infection and inflammation. Further research is required to investigate potential variations in HBP levels in different pediatric conditions.

This study had some limitations. First, it was a single-center, retrospective study with a relatively small sample size. Therefore, a comparison of biomarkers between patients with sepsis and septic shock and those with sepsis but without septic shock was not performed because of the limited number of children with severe sepsis. Thus, these findings might not be applicable to all critically ill children and should be confirmed in large-scale, prospective investigations. Second, the data were obtained at one point in time (admission) without serial measurements. Third, most of the children with severe CAP were administered antibiotics prior to admission, and the effects of this antimicrobial therapy on candidate biomarkers were not evaluated. Finally, there were no data available related to long-term mortality in this study. Therefore, further investigations are necessary to characterize the application of HBP to pediatric populations.

In conclusion, this retrospective, observational, clinical study revealed that plasma levels of HBP are markedly increased in critically ill children with severe CAP at admission to the ICU. This increased HBP level was correlated with severe sepsis and was independently associated with the development of respiratory failure. Therefore, HBP might be a better predictor of disease progression in children with severe CAP than conventionally used biomarkers. More prospective clinical studies are required to confirm our results.

## Data Availability Statement

The raw data supporting the conclusions of this article will be made available by the authors, without undue reservation.

## Ethics Statement

The studies involving human participants were reviewed and approved by the Ethics Committee of Hunan Children's Hospital. Written informed consent to participate in this study was provided by the participants' legal guardian/next of kin.

## Author Contributions

LM and CH contributed to conception and design of the study. CH, CZ, and JZ performed the data collection. LZ and YM carried out the statistical analysis. CH wrote the first draft of the manuscript. LM, CZ, and JZ wrote sections of the manuscript. All authors contributed to revision and approved the final manuscript.

## Funding

This work was funded by Changsha Science and Technology Bureau Science Foundation of China [Grant Number kq2014186].

## Conflict of Interest

The authors declare that the research was conducted in the absence of any commercial or financial relationships that could be construed as a potential conflict of interest.

## Publisher's Note

All claims expressed in this article are solely those of the authors and do not necessarily represent those of their affiliated organizations, or those of the publisher, the editors and the reviewers. Any product that may be evaluated in this article, or claim that may be made by its manufacturer, is not guaranteed or endorsed by the publisher.
